# Recent findings and future directions in photosynthetic hydrogen evolution using polypyridine cobalt complexes

**DOI:** 10.1039/d2dt00476c

**Published:** 2022-04-14

**Authors:** Federico Droghetti, Fiorella Lucarini, Alessandra Molinari, Albert Ruggi, Mirco Natali

**Affiliations:** a Department of Chemical, Pharmaceutical, and Agricultural Sciences (DOCPAS), University of Ferrara Via L. Borsari 46 44121 Ferrara Italy mirco.natali@unife.it; b Département de Chimie, Université de Fribourg, Chemin du Musée 9 1700 Fribourg Switzerland albert.ruggi@unifr.ch; c Centro Interuniversitario per la Conversione Chimica dell'Energia Solare (SolarChem) sez. di Ferrara Via L. Borsari 46 44121 Ferrara Italy

## Abstract

The production of hydrogen gas using water as the molecular substrate currently represents one of the most challenging and appealing reaction schemes in the field of artificial photosynthesis (AP), *i.e.*, the conversion of solar energy into fuels. In order to be efficient, this process requires a suitable combination of a light-harvesting sensitizer, an electron donor, and a hydrogen-evolving catalyst (HEC). In the last few years, cobalt polypyridine complexes have been discovered to be competent molecular catalysts for the hydrogen evolution reaction (HER), showing enhanced efficiency and stability with respect to previously reported molecular species. This perspective collects information about all relevant cobalt polypyridine complexes employed for the HER in aqueous solution under light-driven conditions in the presence of Ru(bpy)_3_^2+^ (where bpy = 2,2′-bipyridine) as the photosensitizer and ascorbate as the electron donor, trying to highlight promising chemical motifs and aiming towards efficient catalytic activity in order to stimulate further efforts to design molecular catalysts for hydrogen generation and allow their profitable implementation in devices. As a final step, a few suggestions for the benchmarking of HECs employed under light-driven conditions are introduced.

## Abstract 2

A collection of cobalt polypyridine complexes employed for the light-driven HER in aqueous solution is presented with the aim of highlighting the relevant chemical motifs that play a role in supporting efficient catalytic activity.

## Graphical abstract

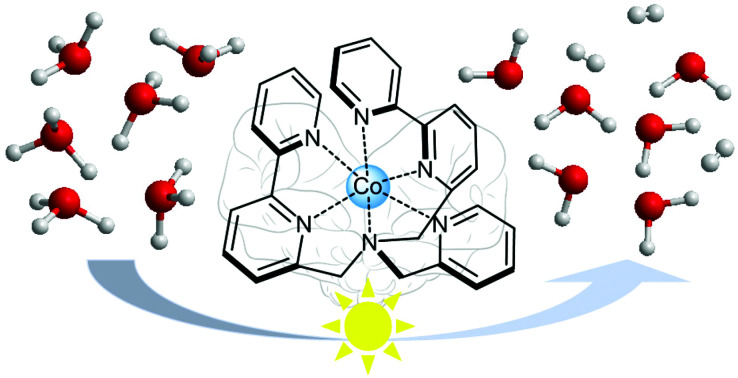

## 1. Introduction

Increasing demand for energy, which is associated with the continuous consumption of fossil fuels, currently presents a serious issue preventing sustainable development.^1^ The present situation clearly requires drastic changes to be consciously undertaken in order to tackle severe phenomena such as global warming and climate change.^2^ One of the possible ways to overcome this problem is to try to progressively shift towards a carbon-neutral energy economy. In this respect, the exploitation of hydrogen as an energy carrier represents a potential solution.^3^ Hydrogen is indeed one of the most abundant elements on Earth, and the combination of hydrogen and oxygen in a fuel cell can yield electricity with water as the only by-product. However, the current production of hydrogen mainly arises from non-renewable sources, *e.g.*, *via* steam-methane reforming. Thus, the real challenge is the production of hydrogen through the exploitation of some renewable resources. In this respect, solar energy represents the most promising and attractive possibility, since it is abundant, evenly distributed, and highly accessible on Earth. The direct conversion of water (protons) into hydrogen is, however, hampered by the complex mechanistic requirements associated with the formation of H–H bonds. A catalyst is indeed necessary to promote the combination of two protons and two electrons into hydrogen molecules at a sufficiently fast rate.

Taking inspiration from the active sites of natural hydrogenases,^4,5^ much effort has been directed in the last few years towards the development of molecular catalysts for the hydrogen evolution reaction (HER) based on Earth-abundant metals. Although molecular species are usually less stable than inorganic materials, molecular catalysts display a high degree of tunability *via* synthetic modification, providing many opportunities for optimization depending on the specific application. Moreover, they also represent discrete, molecular models of the active sites of bulk materials, allowing for detailed mechanistic insight into the catalytic process during the HER when using these materials. Examples of molecular catalysts are coordination complexes based on iron,^6–9^ nickel,^10–13^ and copper.^14–16^ Within this framework, cobalt complexes have so far played a prominent role as molecular catalysts for the HER under both electrochemical and light-driven conditions.^17–20^ Many photochemical systems have been developed using particularly cobaloximes and cobalt diimine-dioximes as cobalt-based molecular catalysts.^21–24^ However, their HER activities are usually only suitable in organic solvents or water/organic mixtures. Furthermore, these complexes suffer from poor stability under high-turnover conditions, which is associated with the hydrogenation of the ligand pool,^22^ preventing the extended application of such molecular systems.

More than ten years ago, the catalytic activities of cobalt polypyridine complexes featuring polydentate ligands were documented.^25^ The main advantages when using this class of compounds are improved stability under operating conditions, thanks to the presence of reductively stable ligands; the absence of decomposition products, such as heterogeneous cobalt nanoparticles (ruled out based on mercury poisoning experiments and dynamic light scattering measurements); and the ability to efficiently perform the HER in a purely aqueous environment.^26,27^ Since then, these encouraging results have led to the preparation of a large variety of cobalt polypyridine complexes through the systematic variation of the molecular design. This includes changing the ligand denticity, the use of pyridine and bipyridine units, the insertion of tertiary amines as coordinating groups, the introduction of electron-donating or -withdrawing substituents, *etc*. Interestingly, most of these compounds have been systematically studied during hydrogen evolution in fully aqueous solution under light-activated conditions.

This perspective aims at evaluating the photo-triggered catalytic activity towards the HER when promoted by polypyridine cobalt complexes in aqueous solution, trying to establish the effects of the molecular architecture on the efficiency and durability of the process. Although several studies of this class of cobalt complexes have been conducted under pure electrochemical conditions^28,29^ or under photochemical conditions when coupled with different molecular components in different solvent mixtures,^19,30–34^ we will limit our discussion to photosynthetic hydrogen evolution performed under homogeneous conditions, using a standard photochemical system involving Ru(bpy)_3_^2+^ (where bpy = 2,2′-bipyridine) as the light-harvesting sensitizer and ascorbate as the sacrificial electron donor. The choice to restrict our analysis solely to a Ru(bpy)_3_^2+^/ascorbate photochemical system stems from the ability to work in pure water (the greenest solvent available) and from the well-established mechanistic aspects associated with the use of this sensitizer/donor pair (see below), giving us the chance to focus our attention on the catalytic aspects of the overall photochemical process. We will start our discussion by examining the general mechanistic pathway of photochemical hydrogen evolution using polypyridine cobalt complexes, then we will give a brief overview of all the catalysts in this class employed under the above-mentioned conditions and set out a structure–activity correlation to identify the most promising catalyst architectures for efficient and durable light-driven hydrogen evolution. Finally, we will present some suggestions aimed at establishing practical guidelines for the possible benchmarking of HER studies under irradiation in order to make photochemical studies as reliable and comparable as possible between different reports.

## 2. Photochemical hydrogen evolution

The ability of polypyridine cobalt complexes to catalyse the reduction of protons to hydrogen stems from their ability to appropriately manage the two-electron/two-proton nature of the HER (eqn (1)) *via* sequences of electron-/proton-transfer processes involving a suitable reducing agent and water:

1 2H^+^ + 2e^−^ → H_2_

In this respect, electrochemical studies performed in organic solution in the presence of a proton donor, often coupled with theoretical calculations, have been employed to elucidate the corresponding mechanistic details.^25,35,36^ Hydrogen evolution using this class of catalyst usually takes place *via* a heterolytic pathway involving either alternating reduction/protonation steps (ECEC mechanism) or two reduction steps followed by two proton transfer processes (EECC mechanism).^37^ The latter pathway is less likely than the former one, as it requires a redox-active ligand, and it is only observed when very weak proton donors are used.^36^ This suggests that, in general terms, hydrogen evolution *via* this class of molecular catalyst in aqueous solution plausibly follows an ECEC mechanism, with water acting as the proton donor. In this scenario, the detachment of a pyridine ligand in the reduced form of the catalyst (usually the Co(i) state) has also been postulated to furnish an internal proton relay to assist the protonation steps *via* intramolecular routes.^36^

**Scheme 1:**
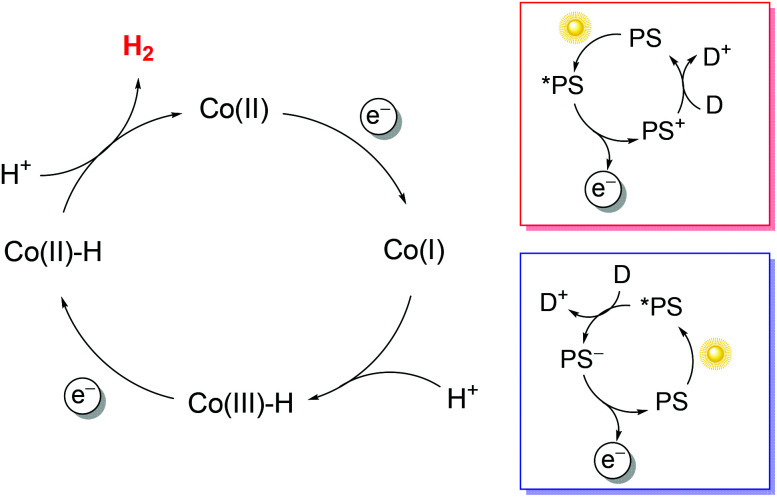
Possible mechanisms for photochemical hydrogen evolution involving cobalt polypyridine complexes (the potential contribution of the polypyridine ligand to protonated intermediates has been neglected). Oxidative (red box) and reductive (blue box) quenching routes are shown. Abbreviations: PS = photosensitizer, D = electron donor.

To exploit polypyridine cobalt complexes as hydrogen evolving catalysts (HECs) under homogeneous photosynthetic conditions, two additional molecular components are required: a light-harvesting sensitizer and an electron donor. Within this three-component system, the reductive activation of the catalyst can be achieved following either an oxidative or a reductive route (Scheme 1). In the former case, the sensitizer, upon excitation, undergoes oxidative quenching by the catalyst, leading to the formation of a reduced catalyst and an oxidized chromophore; the latter then recovers to its initial state upon reaction with the donor (Scheme 1, red box). In the second case, the excitation of the sensitizer is followed by reductive quenching by the donor, leading to a reduced chromophore that eventually undergoes electron transfer to the catalyst (Scheme 1, blue box). *Via* repeating the same reaction sequence a second time, two electrons can be stored on the catalyst platform which is then able to promote proton reduction to a hydrogen molecule. Since the efficiency of the overall photochemical reaction is limited by short-circuiting pathways, *e.g.*, involving detrimental charge recombination, a convenient strategy for studying the HER without such kinetic limitations is the use of a sacrificial electron donor, *i.e.*, a species that undergoes a fast and irreversible reaction upon electron transfer.^38^

**Fig. 1:**
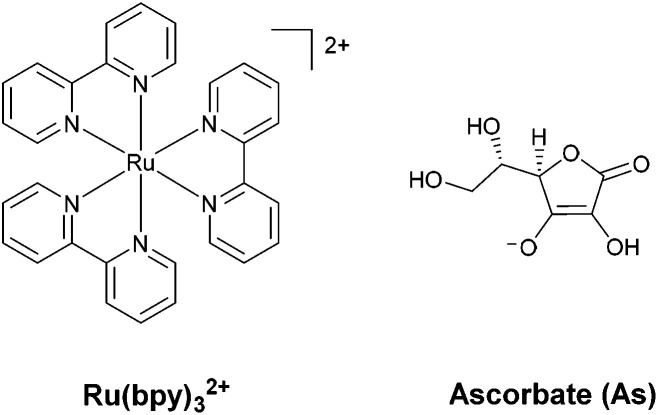
The molecular structures of the Ru(bpy)_3_^2+^ photosensitizer and the ascorbate sacrificial electron donor used in combination with cobalt polypyridine complexes for light-driven hydrogen evolution studies.

Regarding the sensitizer employed for studying photochemical hydrogen evolution with polypyridine cobalt complexes, Ru(bpy)_3_^2+^ (Fig. 1) is usually considered as the molecule of choice as it combines several key properties:^39^ (i) strong absorption in the visible region; (ii) a long excited-state lifetime to efficiently partake in bimolecular reactions; (iii) suitable reduction and oxidation potentials in the excited state (−0.86 and +0.84 V *vs.* SCE in aqueous solution, respectively)^39^ to effectively promote photoinduced electron-transfer processes, either with the catalyst or the sacrificial donor (oxidative or reductive pathway, respectively, see Scheme 1); and (iv) suitable ground-state reduction and oxidation potentials (−1.28 and +1.26 V *vs.* SCE, respectively),^39^ favouring catalyst activation or donor oxidation (reductive or oxidative pathway, respectively, Scheme 1).

As for the sacrificial donor, although many molecules are potentially available,^38^ ascorbate (Fig. 1) has long been employed as a sacrificial agent (oxidation potential of +0.46 V *vs.* SCE)^40^ in combination with Ru(bpy)_3_^2+^ as a sensitizer and a cobalt polypyridine complex as a HEC. This is motivated by the fact that: (i) ascorbate can work at acidic pH levels, where the HER displays thermodynamic and kinetic advantages; (ii) at the typical optimum pH for photochemical hydrogen evolution (*i.e.*, between 4–6), it can be used both as a sacrificial donor and as a buffer (ascorbic acid has a p*K*_a_ value of 4.1 in water); and (iii) it is highly water soluble, allowing the use of high concentrations.

In typical light-driven hydrogen evolution experiments involving the molecular components previously described, a constant concentration of Ru(bpy)_3_^2+^ sensitizer is typically used (between 0.1 and 0.5 mM), which allows for the substantial absorption of incoming light. On the other hand, variable concentrations of catalysts and donors have been employed: values ranging from 1–100 μM are usually reported for cobalt polypyridine complexes, whereas much larger concentrations (in the range of 0.1–1.1 M) have been considered for the ascorbate donor. We must now take into account that bimolecular processes are involved under homogeneous photochemical conditions (Scheme 1). Oxidative photoinduced electron transfer between the excited state of the Ru(bpy)_3_^2+^ sensitizer and the cobalt complex (when experimentally observed) usually occurs with a bimolecular rate constant of around 10^9^ M^−1^ s^−1^,^41,42^ while the rate constant for reductive electron transfer involving the ascorbate donor is ∼10^7^ M^−1^ s^−1^ in aqueous solution.††The electron transfer rate between excited *Ru(bpy)_3_^2+^ and ascorbate is known to be pH dependent since the reaction involves the deprotonated ascorbate anion, whose speciation is related to a p*K*_a_ value of 4.1.^43,44^ The value provided here is related to pH values above 4, which are those typically employed for photochemical studies involving cobalt polypyridine complexes. According to these data, pseudo-first-order rate constants in the range of 10^3^–10^5^ s^−1^ can be estimated for the oxidative quenching of *Ru(bpy)_3_^2+^ by the cobalt complex,‡‡This value refers to the first electron transfer event involving the HEC (Scheme 1). Also assuming oxidative quenching for the second reduction of the HEC, the effective rate is expected to be far slower due to the low concentration of the one-electron reduced and protonated intermediate. while values of >10^6^ s^−1^ are expected for the reductive quenching of *Ru(bpy)_3_^2+^ by ascorbate. The more than ten-fold higher value of the effective rate constant for reductive quenching compared with that for the alternative oxidative quenching pathway thus establishes that light-driven hydrogen evolution in a three-component system involving a cobalt polypyridine complex as the HEC, Ru(bpy)_3_^2+^ as the sensitizer, and ascorbate as the donor unavoidably follows a reductive quenching route (Scheme 1, blue box). In this respect, time-resolved absorption spectroscopy has been employed to monitor the photoinduced electron-transfer sequence related to the first step involving the HEC (namely light-triggered Co(ii) → Co(i) reduction, Scheme 1), and it has been shown that the electron transfer process between photogenerated Ru(bpy)_3_^+^ and the cobalt polypyridine complex is usually fast, with a bimolecular rate constant close to the diffusion-controlled regime (∼10^9^ M^−1^ s^−1^).^41,45,46^

**Fig. 2:**
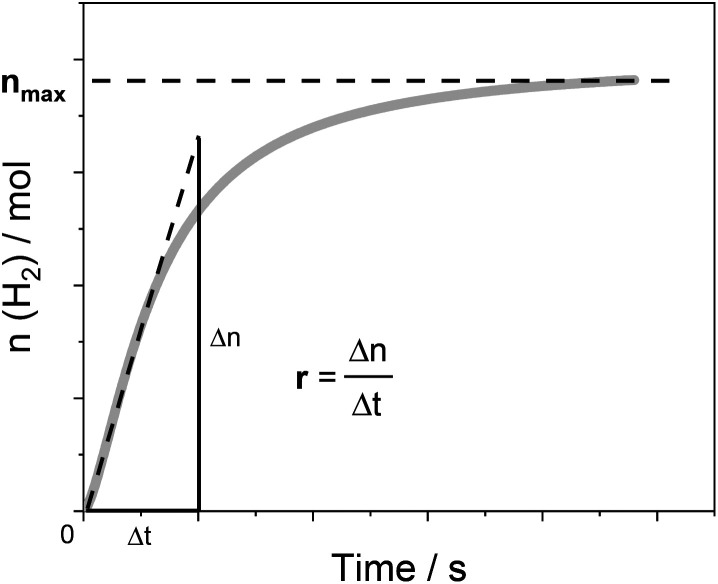
An example of the kinetic trace obtained from a typical photochemical experiment with the identification of two experimental quantities, *r* and *n*_max_, which can be used (according to eqn (2)–(4)) to determine the relevant *figures-of-merit* required for a meaningful comparison of the photosynthetic activities of different cobalt polypyridine complexes in combination with Ru(bpy)_3_^2+^ as the sensitizer and ascorbate as the electron donor.

Below, we will describe the various types of cobalt polypyridine complexes reported in the last few years in combination with Ru(bpy)_3_^2+^ as the sensitizer and ascorbate as the sacrificial agent. The corresponding photosynthetic activities will be examined *via* evaluating key *figures-of-merit* from the photochemical process, which can be extracted based on analysis of the kinetic trace plotting the amount of hydrogen produced in moles *vs.* the irradiation time (Fig. 2). These figures-of-merit include: (i) the initial rate of hydrogen production (*r*, mol s^−1^, Fig. 2); (ii) the derived value of the maximum turnover frequency (TOF, s^−1^), estimated according to eqn (2), where *n*_cat_ is the moles of catalyst in the photolyzed solution; and (iii) the quantum yield (*Φ*) of hydrogen evolution, calculated according to eqn (3), where *φ* is the incident photon flux (in *E* s^−1^ nm^−1^, where *E* = Einstein = 1 mol of photons) and LHE is the light-harvesting efficiency:§§Although common practice defines this quantity as the “quantum yield” of hydrogen evolution, this term should be preferably named “quantum efficiency” (QE).^47^ This latter term refers to the ratio between the measured quantum yield and the theoretical maximum value, with the measured quantum yield being rigorously defined^48^ as the ratio between the moles of product formed (in our case H_2_) and the Einstein (moles of photons) absorbed. Since hydrogen formation is a two-photon process, a theoretical maximum value of 0.5 is expected for the quantum yield.

2 TOF = *r*/*n*_cat_

3
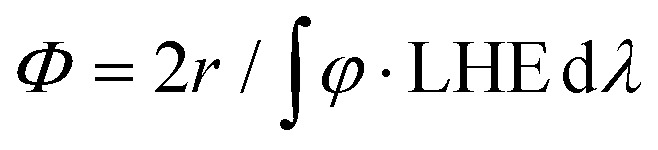


All these parameters are associated with the efficiency of the photochemical reaction. According to the mechanistic picture described above, differences in this regard are expected to be mainly ascribable to the efficiency of the catalyst reduction steps (related to the yields of both the reductive quenching of *Ru(bpy)_3_^2+^ by ascorbate and electron transfer from Ru(bpy)_3_^+^ to both Co(ii) and Co(iii)–H; Scheme 1, blue box) and the ability of the catalyst platform to facilitate the formation of protonated intermediates and the elimination of hydrogen molecules.

Finally, the last *figures-of-merit* that will be considered in this analysis are: (iv) the overall amount of hydrogen produced (*n*_max_, in mol, Fig. 2); and (v) the associated maximum TON value estimated from eqn (4):

4 TON = *n*_max_/*n*_cat_

These values should, in principle, be limited by the consumption of the ascorbate sacrificial donor, as one molecule of hydrogen is photochemically produced per one molecule of reacted ascorbate (which leads to the formation of one molecule of dehydroascorbate through the disproportionation of the corresponding one-electron oxidation product).^38^ Practically, these data are mainly determined by both the sensitizer and catalyst stability, as both components are potentially affected by side-reactions occurring in competition with the profitable forward light-triggered electron-transfer processes that lead to hydrogen elimination (Scheme 1). The maximum TON is usually (*but not always correctly!*) employed in the current literature to describe the overall activity and to measure and compare the efficiencies of photosynthetic systems for hydrogen production. In this respect, although this parameter is intrinsically related to the efficiency of the photochemical process (since fast and efficient pathways will limit the competition of undesired parasitic reactions), it should be better emphasized that this quantity gives mainly information about the stability and durability of a photosynthetic system.

## 3. Cobalt polypyridine complexes

### 3.1. Historical perspective

Polypyridyl cobalt complexes have recently become very popular catalysts for light-driven hydrogen generation. Despite this apparent novelty, the discovery of their activity towards the HER is not so new. As a matter of fact, in 1981, Sutin and co-workers reported early attempts to use cobalt polypyridyl complexes as catalysts for the reduction of protons *via* mixing a Co(ii) salt, bipyridine, Ru(bpy)_3_^2+^, and ascorbate in aqueous solution at pH 5. The photochemical evolution of H_2_ was indeed observed, and it was suggested that Co(bpy)_*n*_^2+^ species were competent molecular systems capable of driving proton reduction. Dihydrobipyridine and dehydroascorbic acid were proposed as possible side products inducing the levelling-off of the light-driven catalytic activity.^49,50^

**Fig. 3:**
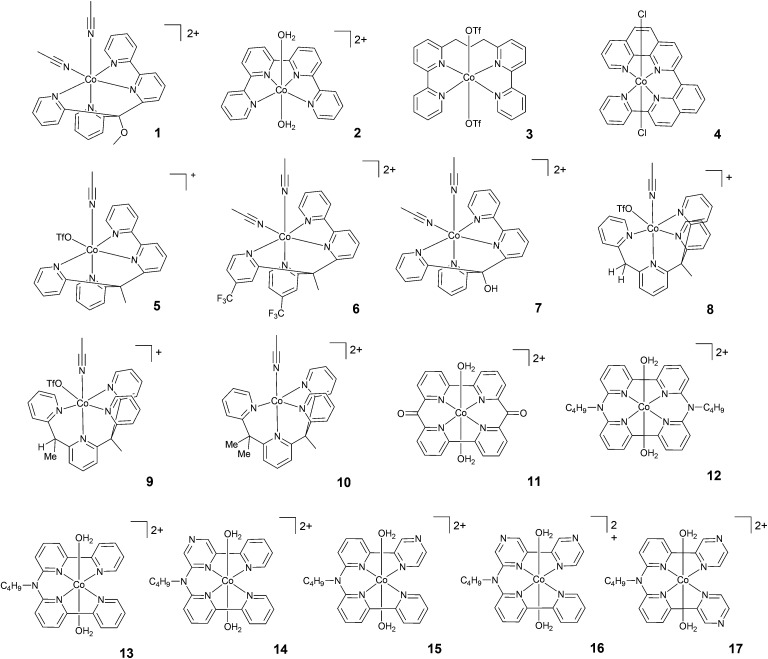
The molecular structures of tetradentate polypyridine complexes used in photochemical hydrogen evolution experiments with Ru(bpy)_3_^2+^ and ascorbate.

Later on, in 2010, Chang and co-workers synthesized a new catalyst based on a tetradentate polypyridyl ligand (1, Fig. 3), which was found to catalyse H_2_ production under electrochemical conditions.^25^ Since that pioneering work, a huge number of new polypyridyl cobalt catalysts have been synthesized and employed as HECs for the reduction of protons. Most of them have also been used in photochemical experiments coupled with Ru(bpy)_3_^2+^ and ascorbate as the sensitizer and sacrificial electron donor, respectively. The use of different pyridyl groups, N donors, connectivities, and substituents has led to a large variety of polypyridine ligand scaffolds and to corresponding mononuclear cobalt complexes. An overview of the most representative cases is presented here. For a clearer description, the polypyridyl cobalt complexes will be grouped into four classes: (i) cobalt complexes based on tetradentate polypyridine ligands; (ii) cobalt complexes based on pentadentate polypyridine ligands; (iii) cobalt complexes based on amino–polypyridyl ligands; and (iv) cobalt complexes based on hexadentate ligands. As previously inferred, all the examples reported here cover complexes that have been employed in light-driven hydrogen evolution studies using Ru(bpy)_3_^2+^ and ascorbic acid as the sensitizer and sacrificial electron donor, respectively. The corresponding *figures-of-merit* from the above-mentioned photochemical cycles are collected in Tables 1–3. The potential of the Co(ii)/Co(i) process measured in an organic solvent is also introduced (when known) as a reference parameter that may help to evaluate the electronic effects of the ligand on redox events associated with the complex and that are relevant to the HER. In this regard, using an organic solvent aims to allow reliable comparison *via* avoiding any potential shift that might occur in aqueous solution as a function of pH due to proton-coupled electron-transfer events. We ought to point out that some cobalt polypyridine complexes have been investigated in the presence of additional electron donors besides ascorbate, namely tris(2-carboxyethyl)phosphine (TCEP).^51–53^ This chemical species can promote the reduction of the dehydroascorbic acid side-product, thus limiting its accumulation during photochemical experiments and consequently improving the maximum TON achievable. As a matter of fact, dehydroascorbic acid can quench the photogenerated Ru(bpy)_3_^+^ reducing agent, leading to a short-circuit in the photochemical reaction.^41^ However, the kinetics of this process are bimolecular in nature and, being dependent on the concentration of dehydroascorbic acid, this process is thus expected to become important only in the later stages of photochemical experiments. Accordingly, the relevant efficiency-related *figures-of-merit*, when estimated during the early stages of photolysis (see above), are not expected to be appreciably influenced upon the addition of a second sacrificial electron donor. Nevertheless, due to the use of an additional chemical species (TCEP), the experimental conditions are intrinsically different from those commonly employed. Hence, for the sake of clarity, the cobalt complexes studied under these specific conditions will not be considered hereafter.

**Table 1:** Relevant data for tetradentate polypyridine complexes used in photochemical hydrogen evolution experiments with Ru(bpy)_3_^2+^ and ascorbate

HEC	*E*/V^a^	[Co]/μM	[Ru]/mM	[As]^b^/M	Buffer^c^	pH	Light	TOF/h^−1^	*Φ*/%	TON	Time/h	Ref.
1	−0.81	20	0.33	0.3	AsB	4.0	LED, 452 nm	73	—	1015	14	25, 46 and 59
2	−0.57^d^	20	0.33	0.3	AsB	5.5	LED, 452 nm	16	—	225	14	46 and 59
3	—	20	0.33	0.3	AsB	5.0	LED, 452 nm	11	—	150	14	46 and 59
4	−0.77^e^	3	0.4	0.3	AsB	4.0	LED, 469 nm	586	—	333	3	54
5	—	20	0.33	0.3	AsB	4.0	LED, 452 nm	134	7.5	1875	14	46 and 59
6	—	20	0.33	0.3	AsB	4.5	LED, 452 nm	30	—	425	14	46 and 59
7	—	20	0.33	0.3	AsB	4.5	LED, 452 nm	76	—	1065	14	46 and 59
8	—	20	0.33	0.3	AsB	5.0	LED, 452 nm	87	—	1565	14	46 and 59
9	—	20	0.33	0.3	AsB	5.5	LED, 452 nm	14	—	260	14	46 and 59
10	—	20	0.33	0.3	AsB	5.0	LED, 452 nm	13	—	235	14	46 and 59
11	—	1	0.5	1.0	AsB	4.0	LED, 453 nm	937	—	1649	24	55
12	−0.65^e^	5	0.5	0.1	AsB	4.5	LED, 455 nm	1220	—	800	4	56
13	−0.69	5	0.5	0.1	AsB	4.5	LED, 455 nm	2070	—	1380	4	56
14	−0.54	5	0.5	0.1	AsB	4.5	LED, 455 nm	450	—	629	4	57
15	−0.57	5	0.5	0.1	AsB	4.5	LED, 455 nm	3419	—	1569	4	57
16	−0.42	5	0.5	0.1	AsB	4.5	LED, 455 nm	376	—	272	4	57
17	−0.48	5	0.5	0.1	AsB	4.5	LED, 455 nm	538	—	324	4	57

a Potential of Co(ii)/Co(i) reduction estimated in acetonitrile and referenced to the saturated calomel electrode (SCE) value.

b [As] = total concentration of ascorbic acid and ascorbate.

c AcB = acetate buffer, AsB = ascorbate buffer, PB = phosphate buffer.

d Taken from ref. 60.

e Measured in DMF.

**Table 2:** Relevant data for pentadentate polypyridine complexes used in photochemical hydrogen evolution experiments with Ru(bpy)_3_^2+^ and ascorbate

HEC	*E*/V^a^	[Co]/μM	[Ru]/mM	[As]^b^/M	Buffer^c^	pH	Light	TOF/h^−1^	*Φ*/%	TON	Time/h	Ref.
18	−1.00^d^	50	0.2	0.1	PB, 1 M	7.0	>455 nm	23	0.23	300	8	58 and 59
19	−1.15^d^	50	0.2	0.1	PB, 1 M	7.0	>455 nm	22	—	290	8	58 and 59
20	−1.31^d^	50	0.2	0.1	PB, 1 M	7.0	>455 nm	10	—	55	8	58 and 59
21	−1.31	50	0.5	0.1	AcB, 1 M	4.0	>400 nm	486	1.7	187	1	41
22	−0.80	20	0.33	0.3	AsB	4.0	LED, 452 nm	660	3.6	1630	13	35
23	−0.74	20	0.33	0.3	AsB	4.5	LED, 452 nm	500	2.7	1390	13	35
24	—	100	0.5	0.1	AsB	5.0	LED, 470 nm	—	—	56	110	51
25	−0.82	20	0.33	0.3	AsB	5.5	LED, 452 nm	—	0.26	190	8	61
26	−0.90	20	0.33	0.3	AsB	5.5	LED, 452 nm	—	0.49	450	8	61
27	−0.78	20	0.33	0.3	AsB	5.5	LED, 452 nm	—	0.10	175	8	61
28	−1.04^e^	2	0.5	0.1	PB, 1 M	7.0	LED, 450 nm	560	—	6900	48	62

a Potential of Co(ii)/Co(i) reduction estimated in acetonitrile and referenced to the saturated calomel electrode (SCE) value.

b [As] = total concentration of ascorbic acid and ascorbate.

c AcB = acetate buffer, AsB = ascorbate buffer, PB = phosphate buffer.

d Measured in CH_2_Cl_2_.

e Recorded for the cobalt analogue with a Cl^−^ axial ligand.

**Table 3:** Relevant data for amino–polypyridine complexes used in photochemical hydrogen evolution experiments with Ru(bpy)_3_^2+^ and ascorbate

HEC	*E*/V^a^	[Co]/μM	[Ru]/mM	[As]^b^/M	Buffer^c^	pH	Light	TOF/h^−1^	*Φ* /%	TON	Time/h	Ref.
29	−1.18^e^	5	0.5	0.1	AcB, 1 M	4.0	LED, 450 nm	1500	∼7	1600	3	63
30	−1.14^e^	5	0.5	0.1	AcB, 1 M	5.0	LED, 450 nm	—	—	1690	3	64
31	−1.30^e^^,^^f^	5	0.5	0.1	AcB, 1 M	4.0	LED, 450 nm	—	—	2770	3	65
32	−1.01^e^^,^^f^	5	0.5	0.1	AcB, 1 M	4.0	LED, 450 nm	—	—	90	3	65
33	−1.23	75	0.5	0.1	AcB, 1 M	5.0	>400 nm	161	0.9	67	3	42
34	−1.19	75	0.5	0.1	AcB, 1 M	5.0	>400 nm	123	0.7	56	3	42
35	−1.22	75	0.5	0.1	AcB, 1 M	5.0	>400 nm	146	0.8	59	3	42
36	−1.22	75	0.5	0.1	AcB, 1 M	5.0	>400 nm	182	1.0	90	3	42
37	−1.24	75	0.5	0.1	AcB, 1 M	5.0	>400 nm	198	1.1	94	3	42
38	−1.46^e^	50	0.1	0.1	AsB	4.0	400–700 nm	—	—	3–15^d^	3	66
38	−1.39^e^	100	0.5	1.1	AsB	4.0	400–700 nm	—	—	59–70	4	67
39	−1.04^e^	50	0.5	0.1	AcB, 1 M	4.0	LED 470 nm	—	—	58^d^	8	45
40	−1.62	100	0.5	0.1	AcB, 1 M	5.0	> 400 nm	7.2	0.05	10	2	68
41	−1.55^e^^,^^f^	5	0.5	0.1	PB, 1 M	6.0	LED, 450 nm	—	—	200	3	69
42	−1.09	1	0.5	0.1	AcB, 1 M	4.0	LED, 475 nm	4020	8.8	2440	4	70
43	−1.11	1	0.5	0.1	AcB, 1 M	4.0	LED, 475 nm	5166	11.3	5520	4	71
44	−1.08	1	0.5	0.1	AcB, 1 M	4.0	LED, 475 nm	4596	10.1	4043	4	71
45	−1.19	1	0.5	0.1	AcB, 1 M	4.0	LED, 475 nm	1782	3.9	1853	4	71
46	−0.89	1	0.5	0.1	AcB, 1 M	4.0	LED, 475 nm	1602	3.5	591	4	71

a Potential of Co(ii)/Co(i) reduction estimated in acetonitrile and referenced to the saturated calomel electrode (SCE) value.

b [As] = total concentration of ascorbic acid and ascorbate.

c AcB = acetate buffer, AsB = ascorbate buffer, PB = phosphate buffer.

d No data explicitly reported, estimated from the available kinetic data.

e Recorded for the cobalt analogue with a Cl^−^ axial ligand.

f Measured in DMF.

### 3.2. Tetradentate polypyridine ligands

A class of polypyridine cobalt complexes widely employed in photochemical hydrogen evolution studies is one based on tetradentate polypyridine ligands. From a structural standpoint, these cobalt complexes achieve a six-coordination structure with two labile ligands, such as counterions or solvent molecules (Fig. 3). 2 was tested under purely aqueous conditions in a study performed by Long, Chang, Castellano, and co-workers.^46^ In the presence of ascorbic acid at pH 5.5, a TON of 225 was achieved after 14 h of irradiation at 452 nm. Furthermore, the same authors synthesized a modified structure (3) with a propylene group between the two bipyridines. The complexes were compared under similar conditions, and 3 achieved a TON of 150. This evidence suggested that the more flexible structure with less distorted square-planar geometry around the metal and better separation of the two π-systems could be responsible for the different activities. Interestingly, this hypothesis was subsequently supported by Thummel and co-workers.^54^ They presented a new catalyst, 4, having a square-planar environment and even more distorted geometry with respect to 2 and 3. 4 was reported to reach a higher TON (*i.e.*, TON = 333) after 3 h of irradiation. A maximum TOF of 586 h^−1^ was also recorded.

Long, Chang, Castellano, and co-workers studied two series of cobalt complexes (1 and 5–7, and 8–10) with the aim of highlighting structure–function relationships.^46^ For the first series, under similar conditions, the following order was found in terms of maximum TONs and TOFs: 5 > 1–7 > 6, showing that the modification of the ligands has significant effects on the catalytic performance. In detail, 6 was less active than 5, suggesting that the introduction of the electron-withdrawing –CF_3_ group significantly decreases the reactivity of the metal centre, possibly slowing down protonation events. Electrochemical investigations have already predicted this trend, as the Co(ii)/Co(i) reduction potential of 5 showed a more positive value, leading to a more stable (less reactive) Co(i) species. Complexes 8–10, with differences in steric hindrance, were investigated under similar conditions in acidic and neutral pH ranges. 8, having lower steric hindrance, showed the highest activity at pH 5, yielding H_2_ with a maximum TON of 1565, whilst 9 and 10 reached maximum TONs of only 260 and 235, respectively. The amount of H_2_ produced by 8 at acidic pH was found to be slightly lower than that produced by 5; by contrast, 8 was more efficient at neutral pH, with a TON of 2400. Alberto and co-workers^55^ reported the hydrogen evolution activity under light-driven conditions of a macrocyclic cobalt(ii) complex featuring two bipyridine units connected *via* two keto bridges (11). At pH 4 and with a catalyst concentration of 1 μM, hydrogen is effectively produced with a maximum TON of 1649 after 24 h of irradiation, with a maximum TOF of 937 h^−1^. Mulfort and co-workers prepared the cobalt complexes 12 and 13, featuring similar tetradentate ligands, connecting two bipyridine units with a nitrogen group in order to insert a potential site for protonation close to the metal centre.^56^ Upon light irradiation of solutions containing 5 μM catalyst in the presence of Ru(bpy)_3_^2+^ and ascorbate, hydrogen production is detected, leading to maximum TONs of 800 and 1380 after 4 h with TOFs of 1220 and 2070 h^−1^ for 12 and 13, respectively. The improved activity in the case of complex 13 was ascribed to its greater structural flexibility. Subsequently, a series of similar complexes (14–17) was prepared featuring redox-active pyrazine groups in place of the pyridine moieties.^57^ Photolysis under comparable conditions as those used for the parent complex 13 yielded hydrogen with maximum TONs of 629, 1569, 272, and 324 and TOFs of 450, 3419, 376, and 538 h^−1^ for 14, 15, 16, and 17, respectively.

### 3.3. Pentadentate polypyridine ligands

**Fig. 4:**
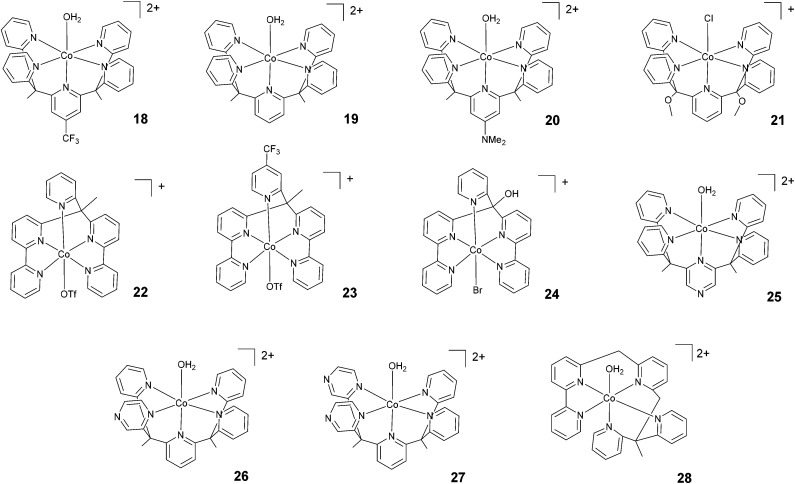
The molecular structures of pentadentate polypyridine complexes used in photochemical hydrogen evolution experiments with Ru(bpy)_3_^2+^ and ascorbate.

Chang, Long, and co-workers investigated the light-driven hydrogen evolution activities of 18–20 (Fig. 4) in aqueous solution. Under the same conditions, 18 displayed the highest TON (*i.e.*, TON = 300) after 8 h of irradiation at pH 7 with a quantum yield of 0.23%.^58^ Conversely, complex 21, synthesized by Iengo, Scandola, and co-workers^41^ and akin to 19 in terms of molecular structure, yielded H_2_ with a TON of 187 during one hour of photolysis at pH 4 with a TOF of 486 h^−1^. Long, Chang, Castellano, and co-workers^35^ subsequently reported 22 and 23 based on bis(bipyridine) and pyridine moieties. 23, bearing a –CF_3_ moiety on the pyridyl group, displayed lower catalytic activity than 22. TONs of 1630 and 1390 and quantum yields of 3.6% and 2.7% were measured for 22 and 23, respectively. Complex 24, based on a distorted octahedral structure akin to those of 22 and 23, was then synthesized by Alberto and co-workers,^51^ and it produced hydrogen upon irradiation with a maximum TON of 56.

Furthermore, Long, Chang, Castellano, and co-workers prepared a series of cobalt complexes (25–27) featuring pentadentate ligands including redox active pyrazines and tested them during light-driven hydrogen evolution.^61^ Complex 26, displaying an equatorial pyrazine, behaves as a better catalyst (maximum TON = 450 and *Φ* = 0.49%) than complexes 25 and 27 (maximum TONs = 190 and 175 with *Φ* = 0.26% and 0.10%, respectively) featuring an axial pyrazine or two equatorial pyrazines, respectively, suggesting that the position and number of non-innocent pyrazine ligands plays a prominent role in determining the catalytic activity.

Very recently, Webster, Zhao, and co-workers reported complex 28 based on a flexible polypyridine ligand,^62^ which is capable of producing hydrogen efficiently at pH 7 in phosphate buffer over 48 h of irradiation in the presence of 0.1 M ascorbic acid, leading to a maximum TON of 6900 with a TOF of 560 h^−1^.

### 3.4. Amino–polypyridyl ligands

**Fig. 5:**
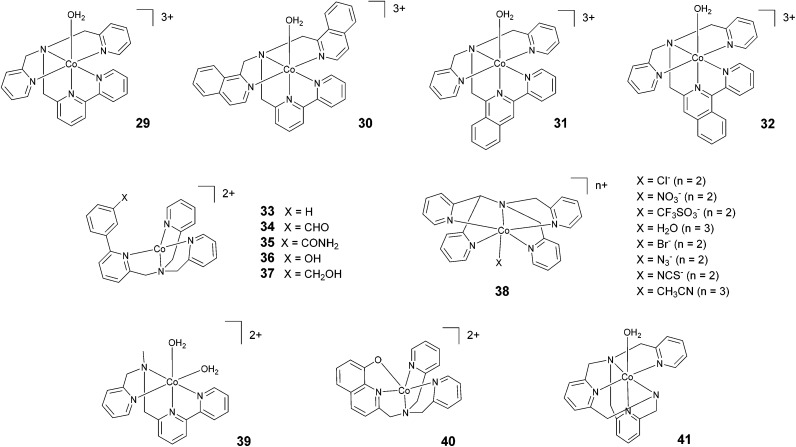
The molecular structures of cobalt catalysts with amino–polypyridyl ligands used in photochemical hydrogen evolution experiments with Ru(bpy)_3_^2+^ and ascorbate.

In 2012, Webster, Zhao, and co-workers reported the synthesis and study of 29 based on a pentadentate ligand containing alkylamino pyridine and bipyridine groups (Fig. 5). The production of H_2_ catalysed by 29 was carried out with a TON of >4000 under optimized conditions, using ascorbic acid and acetate buffer at pH 4 and 3 h of irradiation at a very low catalyst concentration (0.5 μM). A TON of >1600 was obtained with 5 μM catalyst.^63^ These promising results inspired the synthesis of 30, where two isoquinoline groups replaced the two pyridyl groups.^64^ Under similar acidic conditions, 29 and 30 were found to achieve similar TONs, *i.e.*, 1600 and 1690, respectively. However, at pH 7, a TON of 830 was achieved with 30. This value is more than two times higher than the TON obtained with 29 (TON = 390). In 2018, Webster and Zhao synthesized 31 and 32*via* introducing isoquinoline groups into the amino–polypyridyl ligand.^65^ Interestingly, in spite of the structural similarity, the two catalysts displayed big differences in catalytic activity, with 31 (TON = 2770) being much more active than both 32 (TON = 90) and the parent catalyst 30 (TON = 1690). Natali, Zonta, and co-workers studied a series of complexes (33–37) with different substituents at the *meta*-position of the phenyl moiety, as shown in Fig. 5. The complexes produced H_2_ upon irradiation at pH 5, achieving TONs in the range of 56–94 upon 3 h of irradiation with TOFs of 123–198 h^−1^, suggesting no remarkable effects on catalysis as a result of the different substituents.^42^

Wang and co-workers investigated^66^ the effects of the apical ligand (X = Cl^−^, NO_3_^−^, CF_3_SO_3_^−^, and H_2_O) in complex 38. The catalytic activity was found to be surprisingly different when the apical ligand was changed, with the complex bearing a chloride being the most active in the series. To explain this behaviour, the authors suggested that during the catalytic cycle the apical ligand remains coordinated to cobalt, whilst one of the Co–N bonds (*i.e.*, one of the pyridines of the ligand) dissociates to allow the formation of a cobalt hydride intermediate *via* intramolecular proton transfer. This was experimentally corroborated based on an apparent increase in the light-driven hydrogen evolution performance when 0.3 M NaCl was added as an external electrolyte.^66^ In contrast, a study performed by Blackman, Collomb, Crowley, and co-workers^67^ on the same catalyst (38) with a different series of apical ligands (X = CH_3_CN, H_2_O, Cl^−^, Br^−^, N_3_^−^, and NCS^−^) showed no significant differences in light-driven catalytic activity, with maximum TONs ranging from 59–70. Conversely to Wang and co-workers,^66^ the authors suggested that the dissociation of the apical ligand is likely to occur before the formation of the cobalt hydride catalytic intermediate.^67^ As a general comment, the different activities shown towards hydrogen evolution measured in the two different labs might partly arise from the different experimental conditions employed (different Ru(bpy)_3_^2+^ and ascorbate concentrations, different irradiation sources, and different reactor geometries).^66,67^ This notwithstanding, while the observation of different hydrogen evolution kinetics (and concomitant maximum TONs) points towards the apical ligand having non-negligible effects on catalysis,^66^ the appreciable narrow ranges of the measured TONs in both instances (see Table 3) probably suggest a non-dominating role.^67^

Ott and co-workers reported the hydrogen evolution activity of the tetradentate amino–pyridyl cobalt(ii) complex 39 in combination with Ru(bpy)_3_^2+^ and ascorbate at pH 4. A maximum TON of 58 was obtained after 8 h of light irradiation.^45^

Complex 40 represents one of the very few examples of a ligand containing O atoms in the coordination sphere, although this ligand architecture did not have beneficial effects on the catalytic performance. Indeed, a maximum TON of 10 and a TOF of 7.2 h^−1^ were recorded in the presence of 1 M acetate buffer (pH 5), ascorbic acid, and 0.1 mM catalyst.^68^ The authors attributed this poor performance to the large reduction potential required to activate HER catalysis when using complex 40, which leads to enhanced sensitizer degradation under the operating conditions. Moderate hydrogen yields (TON = 200) were also achieved^69^ using complex 41 at pH 6.

### 3.5. Hexadentate ligands

**Fig. 6:**
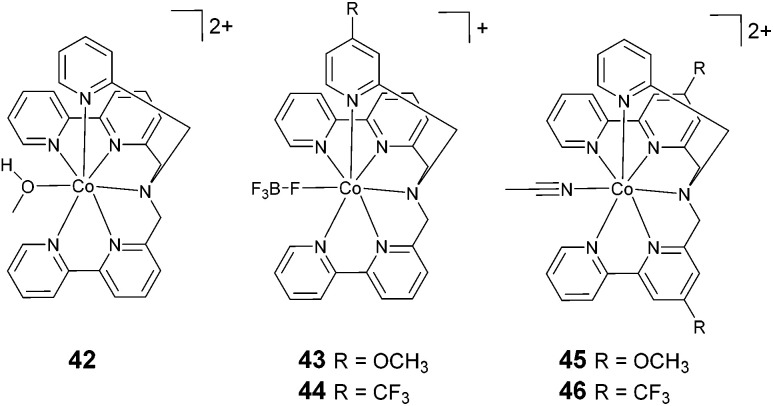
The molecular structures of cobalt catalysts with hexadentate ligands used in photochemical hydrogen evolution experiments with Ru(bpy)_3_^2+^ and ascorbate.

The exploration of catalysts based on hexadentate ligands (Fig. 6) is very recent. In fact, the utilization of such ligands is quite counterintuitive, since the presence of a free coordination site is usually considered an essential requisite for HER catalysis, as this provides a suitable platform for proton anchoring and the subsequent formation of a cobalt-hydride intermediate. However, such complexes often show surprisingly high catalytic efficiencies. The first example of a cobalt complex featuring a hexadentate ligand (42, Fig. 6) was reported by Ruggi and co-workers.^70^ Interestingly, in this compound, the cobalt centre shows an exotic heptacoordinated environment, with one coordination site occupied by a labile ligand. Upon the blue-light irradiation of an aqueous solution containing 1 μM 42, Ru(bpy)_3_^2+^, and ascorbate, hydrogen is produced with a maximum TON of 2440, a TOF of 4020 h^−1^, and *Φ* = 8.8%, showing remarkable efficiency and stability when compared to previously reported polypyridine cobalt complexes. DFT computational studies evidenced that the pyridine ligands can detach from the metal centre during the catalytic cycle, providing internal proton relays that assist hydrogen formation.^36^

A systematic structure–activity relationship study was then performed in order to shine light on the catalytic mechanism.^71^ The introduction of substituents to the pyridine moiety (complexes 43 and 44) leads to an increase in the catalytic activity under photochemical conditions with respect to the archetypal unsubstituted compound (42). Conversely, the introduction of the same groups to the bipyridine moiety (complexes 45 and 46) results in a decrease in the catalytic efficiency. Remarkably, the chemical nature of the group (*i.e.*, electron withdrawing or electron donating) has a less prominent role on the catalytic efficiency. At pH 4 (1.0 M acetate buffer) in the presence of ascorbate and Ru(bpy)_3_^2+^ at a catalyst concentration of 1 μM, a TON of up to 5520 was achieved using complex 43, with a TOF of up to 5166 h^−1^ and a quantum yield of 11.3%. Furthermore, DFT calculations suggested that the introduction of substituents to the pyridine ring could open up new, thermodynamically favourable pathways to accelerate the proton transfer processes in an intramolecular fashion.^36,71^

## 4. Structure–activity correlation

In the present discussion we aim to interpret the data reported above in order to attain specific guidelines which might help to highlight the most promising chemical structures for achieving efficient and durable photochemical hydrogen evolution. We should emphasize that the *figures-of-merit* collected above for different catalysts (Tables 1–3) have been obtained by different research groups using different photochemical setups. In particular, variable reaction volumes and different irradiation sources (Xe lamps with variable cut-offs, monochromatic LEDs at different wavelengths, *etc*.) and reactor geometries have been employed. Accordingly, the following comparisons should be considered with an appreciation of the error that may arise from the diverse experimental conditions used in the different studies. In this regard, when specific systematic conclusions are attempted, comparisons between data obtained under similar conditions are privileged.

### 4.1. Ligand structure and denticity

The cobalt polypyridine complexes employed in light-driven hydrogen evolution display a large variety of chemical motifs as far as the ligand architecture is concerned. When it is attempted to make a general comparison between different classes, cobalt complexes featuring amino–polypyridyl ligands (Fig. 5, 6 and Table 3) usually show improved performance in terms of their light-driven catalytic efficiencies (maximum TOFs and quantum yields) with respect to molecular analogues having just pyridine in the ligand framework. This observation can be possibly ascribed to the enhanced flexibility of the ligand imparted by the presence of a tertiary amine group, which allows the complex to easily adapt its molecular structure to the relevant intermediates formed along the catalytic cycle towards hydrogen elimination. Interestingly, comparable performances as those obtained using such amino–polypyridyl cobalt complexes can also be attained when using polypyridine-only analogues featuring flexible ligands, such as complexes 13, 15, 22, 23, and 28.^35,56,57,62^

However, it is worth pointing out that the improved activities of complexes featuring amino–polypyridyl ligands are particularly evident for complexes displaying high ligand denticity (catalysts 29–31 and 42–46).^62–65,70,71^ As a matter of fact, modest performances have been shown by corresponding complexes featuring tetradentate ligands (compounds 33–37 and 39).^42,45^ The moderate activities of these latter complexes might be associated with deviation from the (pseudo)octahedral geometry typically encountered in polypyridine cobalt complexes. It is indeed likely that the resulting 3D structure and associated electronic configuration might not be as optimal for the stabilization of cobalt hydride intermediate(s), which is required to promote hydrogen formation. For instance, trigonal bipyramidal geometry is expected for cobalt complexes with tetradentate tris(2-pyridylmethyl)amine-type ligands.^72^ On the other hand, the largest TOFs and quantum yields within the amino–polypyridyl series have been measured using cobalt complexes featuring hexadentate ligands (42–46).^70,71^ For these catalysts, beside the favourable effects arising from ligand flexibility, the ability of the same amino–pyridyl platform to assist the required proton transfer events *via* intramolecular pathways involving detached pyridines appears key to the achievement of high catalytic rates.^36^ A similar effect has also been suggested to explain the superior performance of the pentacoordinate complex 26, containing a pyrazine in an equatorial position, with respect to the analogue 25, bearing a pyrazine in an axial position.^61^

A comparison of the light-driven hydrogen evolution activities of amino–polypyridyl cobalt complexes clearly suggests that the inherent advantages gained from having a flexible ligand must be properly matched with corresponding structural effects and the resulting coordination environment around the cobalt centre. Similar considerations can be made to explain the variable trends in the light-driven catalytic activities shown by tetradentate polypyridine complexes (Fig. 3 and Table 1) and their pentadentate analogues (Fig. 4 and Table 2). In the latter case, however, electronic effects arising from the presence of redox-active ligands and the introduction of electron-withdrawing or -donating substituents have to be considered in more detail to rationalize the corresponding catalytic activities (see below).

The great effort made to synthesize tetradentate polypyridine ligands and the resulting cobalt complexes (Fig. 3 and Table 1) has allowed deep insight to be gained into the relevant structural motifs that play a key role in promoting efficient hydrogen evolution under photochemical conditions. Experimental evidence interestingly suggests that: (i) tetradentate ligands that leave two *cis* coordination sites unused yield considerably better catalysts than tetradentate ligands leaving *trans* coordination sites free (1, 5, 7, and 8*vs.*2–4, Fig. 3 and Table 1);^46,54^ (ii) an increase in the steric hindrance of the tetradentate ligand decreases the activity of the corresponding cobalt complex (5 > 1 > 7 and 8 > 9 > 10),^46^ supporting the hypothesis previously envisioned that a large deviation from (pseudo)octahedral geometry is detrimental to efficient catalysis; (iii) chelating macrocyclic ligands yield highly active catalysts (11–17); (iv) in examples utilizing macrocycles, the catalytic efficiencies depend on the presence/absence of open positions (*e.g.*, 12*vs.*13) and the structural distortion of the resulting cobalt complex;^55–57^ and (v) the introduction of non-coordinating groups capable of hydrogen bonding, thus possibly acting as intramolecular proton shuttles, does not seem to be a valuable strategy for improving the catalytic activity, as proved by complexes 33–37.^42^

Although structural features such as ligand flexibility and distortion are relevant characteristics impacting the efficiency of light-driven hydrogen evolution (*i.e.*, the maximum TOF and quantum yield), other chemical motifs have to be considered to account for the observed trend in the maximum TONs. As previously outlined, the achievement of a large TON is strictly related to the stability of both the sensitizer and the catalyst. Ru(bpy)_3_^2+^ is indeed known to undergo progressive decomposition during continuous photolysis. This involves, on one hand, the population of the triplet d–d state (^3^MC) from the triplet metal-to-ligand charge transfer (^3^MLCT) excited state in competition with reductive quenching by the ascorbate donor, leading to the decoordination of one bpy ligand and the coordination of ascorbate or buffer anions.^46,63*a*^ On the other hand, the deactivation of the sensitizer might also occur at the reduced Ru(bpy)_3_^+^ level in competition with electron transfer from the latter species to the HEC.^49,50,73^ When it comes to possible decomposition pathways involving cobalt polypyridine complexes, these have been observed to involve the decoordination of the metal from the ligand and the consequent loss of cobalt ions in solution,^62,74^ likely occurring from the Co(i) oxidation state of the complex, which usually displays lower coordination numbers and longer Co–N bonds.^36,71^ In this respect, possible solutions for achieving large TONs should involve the following considerations: (i) the use of cobalt polypyridine complexes featuring ligands with high denticity (hexadentate > pentadentate > tetradentate); (ii) within the same class of catalyst, the use of stronger-ligand-field bipyridines over pyridines (see, *e.g.*, 5*vs.*8 for tetradentate complexes, 22, 23, and 28*vs.*18–20 for pentadentate examples, and 29–31*vs.*33–41 for amino–polypyridyl complexes); and (iii) the exploitation of chelating macrocyclic ligands (11–17).^55–57^ Interestingly, the observation^70,71^ of the largest maximum TONs when using complexes with hexadentate ligands (42–46, Fig. 6 and Table 3) is fully consistent with this notion.

### 4.2. Pyridine *vs.* bipyridine

When each single class of catalyst is examined in detail, two subcategories can be easily distinguished, namely polypyridine and amino–polypyridyl cobalt complexes featuring either separated (*i.e.*, electronically decoupled) or linked (*i.e.*, electronically coupled) pyridine. Specifically, bipyridines are usually employed in the construction of polydentate ligands with the aim of allowing ligand-based redox events close to the Co(ii)/Co(i) couple. This situation can indeed help to stabilize the energies of relevant catalytic intermediates required for effective hydrogen generation, thus allowing for the potential acceleration of the catalytic routine and the consequent improvement of the activity under light-assisted conditions. Furthermore, as previously pointed out, the improved chelating abilities and ligand-field strength of the bipyridine ligand over single pyridine can possibly enhance the stability of the catalytic platform against ligand dissociation under high-turnover conditions.^62,74^

Consistent with expectations, the introduction of a bipyridine group into the ligand framework turns out to be effective at enhancing the catalytic activity both in terms of efficiency and durability for all selected classes of cobalt complexes. For tetradentate polypyridine catalysts, the insertion of a bipyridine unit into the ligand of complex 5 leads to both a higher TOF and TON than those observed for the structurally similar complex 8 featuring a methylene linker between the two pyridines (Fig. 3 and Table 1).^46^ Furthermore, the presence of two bipyridine units in the chelating macrocyclic ligands of complexes 11–17 is presumably responsible for the highest performances being shown by these examples within the class of tetradentate complexes.^55–57^ A similar trend is also seen when pentadentate polypyridine catalysts are considered. As a matter of fact, complexes featuring a bipyridine unit (22 and 23) clearly perform better than their corresponding molecular analogues with only a single pyridine (18–20).^35,58,59^ Similar conclusions can be finally drawn when comparing the photocatalytic activities of cobalt complexes with amino–polypyridyl ligands, where the presence of bipyridine (*e.g.*, in complexes 29–31 and 42–46)^63–65,71^ leads to both larger TOFs and TONs than the related complexes without a bidentate moiety (*e.g.*, 33–41).^42,45,66–69^

### 4.3. Electronic effects

The variation of the electronic properties of a complex is usually achieved upon the modification of the electronic density of the ligands *via* the introduction of suitable substituents. However, only a very limited number of reports has been published so far concerning the structure–activity relationship in cobalt-based catalysts for light-triggered hydrogen production. Systematic structure–activity studies, in particular, are extremely rare. One of the very few examples of such a study concerns the family of pentadentate polypyridine complexes 18–20 (Fig. 4), in which an electron-withdrawing group (EWG) or electron-donating group (EDG) was introduced to the axial pyridine.^58^ According to this study, the introduction of an EWG like –CF_3_ (18) slightly improves the performance of the catalyst with respect to the pristine non-substituted complex 19. Conversely, the introduction of an EDG like –N(CH_3_)_2_ (20) induces a pronounced decrease in activity. However, the opposite behaviour was reported when comparing the activities of complexes 5 and 6 and the activities of 22 and 23. In these cases, the introduction of an EWG group to the pyridine moiety induces a decrease in both catalytic efficiency and durability with respect to the pristine unsubstituted complex.^35,46^ A recent systematic study of the hexadentate complexes 42–46 (Fig. 6 and Table 3) helped to clarify the reason for these apparently differing observations.^71^ In particular, it was observed that the position of the substituent plays a more prominent role than the electronic nature. In fact, the introduction of either an EWG or an EDG to the pyridine ring induces an increase in catalytic efficiency. Conversely, the introduction of the same groups to the bipyridine moiety induces a decrease in efficiency with respect to the unsubstituted compounds. This behaviour is attributed to the modification of the thermodynamic and kinetic parameters that are relevant to HER catalysis. More specifically, the introduction of a substituent to pyridine, regardless of its electronic character, induces the more favourable detachment of this unit, thus furnishing an internal proton transfer relay that can accelerate hydrogen elimination.^71^

The replacement of pyridine units with redox-active pyrazine or isoquinoline moieties has also been explored as an alternative method for tuning the electronic properties. In the case of complexes bearing isoquinolines (30–32),^64,65^ higher performance has been observed with respect to the pristine compound 29.^63^ This improvement has been attributed to the stabilization of the low-valence cobalt redox states, supported by the extended conjugation of the isoquinoline ligand.^64,65^ In fact, complex 32, showing reduced conjugation because of steric hindrance, is less active than complex 31. As for the use of pyrazines, in general terms, their introduction seems to be a not-so-favourable strategy for the achievement of efficient and stable catalysis.^57,61^ As a matter of fact, the involvement of the redox-active ligand in the reduced state of the catalyst might have detrimental effects on the light-driven HER, mainly arising from the poor stability of the cobalt complex (see, *e.g.*, complexes 16 and 17 and 25 and 27).^57,61^ On the other hand, as previously mentioned, the introduction of a single pyrazine at the equatorial position of the pentadentate complex 26 turned out to be beneficial.^61^ Hence, analogously to what has been observed when EWGs and EDGs are introduced as substituents, in the case of isoquinoline- and pyrazine-substituted catalysts, the location of the group, rather than their intrinsic electronic effects, seems to play a crucial role (see comparisons of 30*vs.*31 and 25*vs.*26).

## 5. Conclusions and perspectives

In this perspective, various cobalt polypyridine complexes employed as catalysts for light-driven hydrogen production, in the presence of Ru(bpy)_3_^2+^ as the sensitizer and ascorbate as the electron donor, have been presented. The main advantages supporting the use of these molecular complexes as HECs are their high activities towards the photoinitiated HER in fully aqueous environments and their enhanced stabilities under high-turnover conditions. Many structurally different analogues have been screened by several research groups all over the world to try to establish the most promising ligand architectures for improved hydrogen evolution. The comprehensive analysis presented herein suggests several hints relating to the required structural characteristics for an efficient HER catalyst. In particular, flexible multidentate ligands capable of adapting to the coordination environment around the cobalt centre during the catalytic mechanism and that can provide internal relays for facilitating proton transfer processes should be preferably considered. In this respect, we strongly recommend mechanistic studies that also evaluate catalytic intermediates featuring protonated ligands rather than just examining metal-centred hydride species. Second, chelating ligands showing either high denticity or macrocyclic structures can provide improved stability under the chosen operating conditions *via* avoiding decomposition pathways involving the decoordination of the cobalt centre. Furthermore, complexes featuring bipyridine moieties display superior performance compared with single pyridine moieties *via* favouring the stabilization of the reduced catalyst intermediates. Finally, electronic effects arising to chemical substituents can play an effective role in modulating the catalytic performance under photoirradiation conditions, provided that specific sites within the polypyridine ligand are properly identified. All these key features should be carefully considered during the design of novel polypyridine cobalt complexes for light-driven hydrogen generation and for the successful application of known molecular catalysts (or their derivatives) in energy conversion schemes.

Finally, we wish to add a few observations of a general nature, which focus on (but are not limited to) the photochemical studies of the HER catalysed by cobalt polypyridine complexes that are reported in the present perspective. As previously highlighted, different and specific *figures-of-merit* should be provided to describe the catalytic performances of molecular catalysts under irradiation conditions in a comprehensive manner, namely maximum TONs, TOFs, and quantum yields, and the experimental conditions under which these data have been obtained should be reported too. As can be observed in Tables 1–3, all these figures are not always provided and, thus, they are not fully available for the cobalt complexes described here. The same is also true for many other molecular HECs reported to date.^75,76^ In particular, the quantum yield of hydrogen formation, which is the most meaningful efficiency-related parameter, is rarely reported. This situation strongly prevents a straightforward comparison of light-driven catalytic performance in general terms. Accordingly, we strongly encourage measurements of this latter quantity when light-driven hydrogen evolution studies and more general photochemical studies are reported.

Furthermore, due to the complex mechanism that is the basis of the light-driven hydrogen production process (Scheme 1) and the variable experimental conditions employed in different labs (the concentrations of reactants, cell shape and volume, light source, irradiation geometry), a global comparison of the photosynthetic performances of molecular complexes is hardly feasible, even assuming that all relevant *figures-of-merit* are known along with the experimental conditions under which they have been extracted. As a matter of fact, under the same photoreaction conditions, even the simple variation of photon flux turns out to influence the resulting outcome in a substantial manner in terms of TONs, TOFs, and quantum yields. This has been documented, *e.g.*, during homogeneous light-driven water oxidation catalysis,^77–79^ which is a similar (on a mechanistic basis) symmetric photochemical transformation to the one discussed in the present perspective. These considerations immediately call for the need to identify specific experimental conditions under which homogeneous photochemical reactions, including (but not limited to) hydrogen production, can be properly compared. As a matter of fact, while the benchmarking of activity is rather well established in the electrocatalysis realm,^80,81^ this is currently missing in photocatalysis.

Based on these considerations, we try to add herein a few suggestions that might help to make results from light-driven hydrogen evolution as reliable and comparable as possible between different labs. For the sake of simplicity, we will limit our discussion to the experimental conditions discussed in the present manuscript, *i.e.*, fully aqueous photochemical systems involving Ru(bpy)_3_^2+^ as the sensitizer and ascorbate as the sacrificial electron donor. (i) As a first requirement, a standard irradiation source should be considered. In this regard, we propose the use of a solar simulator (AM 1.5G) calibrated at a power of 1 sun (100 mW cm^−2^). The use of any cut-off filter must then be specified. (ii) A limited reaction volume (ideally below 10 mL) should be considered for screening tests. The entire reaction solution should be irradiated, and a constant temperature (preferably in the range of 15–25 °C) should be kept during photolysis experiments (temperature has indeed been observed to considerably affect the photochemical HER activity).^46,70^ (iii) Reactors featuring flat walls (akin to a standard cuvette) should be employed in order to avoid any loss of irradiation light through scattering phenomena. (iv) Fixed quantities of both sensitizer and donor should be employed. We suggest concentrations of 0.5 mM and 0.1 M for Ru(bpy)_3_^2+^ and ascorbate, respectively, allowing for the substantial absorption of incoming light and a suitable quenching yield for the photogeneration of the reduced sensitizer. (v) The regulation of pH (which has a remarkable role in determining the catalytic activity^43,44^ and also impacts the buffering capacity of the reaction medium) should be performed by means of the use of an external inert electrolyte (*e.g.*, acetate for acidic conditions and phosphate for neutral conditions). (vi) Akin to the approach adopted in the dye-sensitized solar cell (DSSC) community,^82^ where results from new dyes are usually benchmarked against the standard N719, photocatalytic tests on new compounds should be compared with those involving a reference molecular catalyst. In this respect, we propose the use of the well-known cobalt complex [Co(CR)Cl_2_]^+^, featuring the tetra(aza)-macrocyclic ligand CR (2,12-dimethyl-3,7,11,17-tetra-azabicyclo(11.3.1)-heptadeca-1(17),2,11,13,15-pentaene), at a fixed concentration (50 μM) as a standard molecular catalyst.^83,84^ This complex can be obtained through well-established synthetic routes,^85^ and it is active towards the light-driven HER under fully aqueous conditions in combination with the Ru(bpy)_3_^2+^ photosensitizer and ascorbate donor.^86,87^ Under 1 sun illumination in the presence of 0.5 mM Ru(bpy)_3_^2+^ and 0.55 M ascorbate at pH 4.1, the cobalt complex [Co(CR)Cl_2_]^+^ at a concentration of 50 μM shows significant performance during the HER, leading to a maximum TON of 680 and a maximum TOF of 324 h^−1^.^87^ These values are perfectly in line with those observed for the cobalt polypyridine complexes described here, thus justifying its possible use as a reference system for the benchmarking of molecular catalysts in future photochemical studies.

Under the photoirradiation conditions suggested here (points i–v) and *via* performing comparison tests with a standard catalyst (point vi), we firmly believe that the determination of maximum TONs, TOFs, and quantum yields for novel molecular HECs will allow the straightforward and meaningful assessment of their intrinsic catalytic abilities. We also think that, with these data in hand, the rational design and subsequent realization of more complex devices for solar-to-hydrogen conversion using cobalt polypyridine complexes, or molecular catalysts in general, will be considerably easier to accomplish.

## Conflicts of interest

There are no conflicts to declare.

## Supplementary Material
